# Temporal reliability of cytokines and growth factors in EDTA plasma

**DOI:** 10.1186/1756-0500-3-302

**Published:** 2010-11-13

**Authors:** Tess V Clendenen, Alan A Arslan, Anna E Lokshin, Annika Idahl, Göran Hallmans, Karen L Koenig, Adele M Marrangoni, Brian M Nolen, Nina Ohlson, Anne Zeleniuch-Jacquotte, Eva Lundin

**Affiliations:** 1 Department of Environmental Medicine, New York University Langone Medical Center, New York, NY, USA; 2 New York University Cancer Institute, New York University Langone Medical Center, New York, USA; 3 Department of Obstetrics and Gynecology, New York University Langone Medical Center, New York, NY, USA; 4 Department of Medicine, University of Pittsburgh Cancer Institute, Division of Cancer Prevention and Population Science, University of Pittsburgh, Pittsburgh, USA; 5 Department of Pathology, University of Pittsburgh, Pittsburgh, USA; 6 Department of Ob/Gyn Reproductive Sciences, University of Pittsburgh, Pittsburgh, USA; 7 Department of Clinical Sciences, Obstetrics and Gynecology, Umeå University, Umeå, Sweden; 8 Department of Public Health and Clinical Medicine/Nutritional Research, Umeå University, Umeå, Sweden; 9 Department of Medical Biosciences, Pathology, Umeå University, Umeå, Sweden

## Abstract

**Background:**

Cytokines are involved in the development of chronic diseases, including cancer. It is important to evaluate the temporal reproducibility of cytokines in plasma prior to conducting epidemiologic studies utilizing these markers.

**Findings:**

We assessed the temporal reliability of CRP, 22 cytokines and their soluble receptors (IL-1α, IL-1β, IL-1RA, IL-2, sIL-2R, IL-4, IL-5, IL-6, sIL-6R, IL-7, IL-8, IL-10, IL-12p40, IL-12p70, IL-13, IL-15, IL-17, TNFα, sTNF-R1, sTNF-R2, IFNα, IFNγ) and eight growth factors (GM-CSF, EGF, bFGF, G-CSF, HGF, VEGF, EGFR, ErbB2) in repeated EDTA plasma samples collected an average of two years apart from 18 healthy women (age range: 42-62) enrolled in a prospective cohort study. We also estimated the correlation between serum and plasma biomarker levels using 18 paired clinical samples from postmenopausal women (age range: 75-86).

Twenty-six assays were able to detect their analytes in at least 70% of samples. Of those 26 assays, we observed moderate to high intra-class correlation coefficients (ICCs)(ranging from 0.53-0.89) for 22 assays, and low ICCs (0-0.47) for four assays. Serum and plasma levels were highly correlated (r > 0.6) for most markers, except for seven assays (r < 0.5).

**Conclusions:**

For 22 of the 31 biomarkers, a single plasma measurement is a reliable estimate of a woman's average level over a two-year period.

## Introduction

Cytokines and growth factors regulate proliferation, apoptosis, and angiogenesis, processes implicated in the development and progression of a number of chronic diseases. Elevated circulating levels of certain inflammation markers, namely C-reactive protein (CRP) and interleukin (IL)-6, have been associated with subsequent risk of cardiovascular disease [1,2] diabetes [3,4], and cancer [5]. Studies investigating the influence of biomarkers on subsequent risk of disease must obtain biological samples collected prospectively to minimize bias due to the influence of existing disease on marker levels. In most studies, prospectively collected samples are obtained from established cohorts, which often have only a single blood sample from each participant. Although basal cytokine and growth factor levels are determined in part by heritability [6,7], they are also likely to be influenced by other factors. Since cytokines and growth factors vary in both acute (e.g. infection, injury, etc.) and chronic inflammatory conditions (e.g. autoimmune disease, obesity, cardiovascular disease, cancer), it is important to determine whether circulating marker levels are reflective only of the short term physiological state or if they represent an individual's average levels over time, relative to other individuals.

The Luminex methodology is well-suited for analyses of a large number of banked samples from prospective cohort studies because it allows for simultaneous measurement of multiple analytes, thereby reducing sample volume requirements, cost, and labor compared to other earlier methods (e.g. single analyte ELISAs)[8]. Our group has previously shown that a number of inflammation markers and growth factors measured using Luminex technology in stored *serum *samples, including IL-1β, IL-1 receptor antagonist (Ra), IL-2, IL-4, IL-5, IL-6, IL-10, IL-12p40, IL-12p70, TNF, soluble TNF-receptor 1 (R1), soluble TNF-R2, CRP, hepatocyte growth factor (HGF), and epidermal growth factor receptor (EGFR) have sufficient temporal reliability to be used for epidemiological studies (intraclass correlation (ICC) ≥ 0.55)[9]. Another group reported similarly moderate to high ICCs (ranging from 0.57-0.89) for *serum *levels of TNF, IL-1β, IL-6, IL-8, IL-10, CRP, and HGF in a study of 48 healthy Chinese men [10]. To our knowledge only one study evaluated variation in a small number of *plasma *cytokines (type of anticoagulant unknown) measured using Luminex and found that temporal reliability was high for IL-1α, IL-4, IL-8, and IL-10, moderate for TNFα, and low for IL-1RA [11]. The purpose of the present study was to evaluate the temporal reliability of a broad range of cytokines and growth factors in *EDTA plasma *samples. We also examined the correlation between serum and plasma cytokines measured using Luminex technology.

## Materials and methods

### Study Design

Study subjects were from the Northern Sweden Health and Disease Study (NSHDS) cohort, which has been described previously [12]. Briefly, since 1985, participants between the ages of 30-70 have been recruited from population-based cardiovascular and/or breast screening programs in Northern Sweden. At enrollment, participants provided 20 mL of fasting peripheral venous blood, drawn with tubes containing EDTA as an anti-coagulant. A second EDTA plasma sample has since been collected from a subset of the cohort. Samples were drawn, processed, and stored under a standardized protocol, in which they were centrifuged immediately after blood draw, and plasma was aliquotted and stored at -80°C.

**Figure 1:**
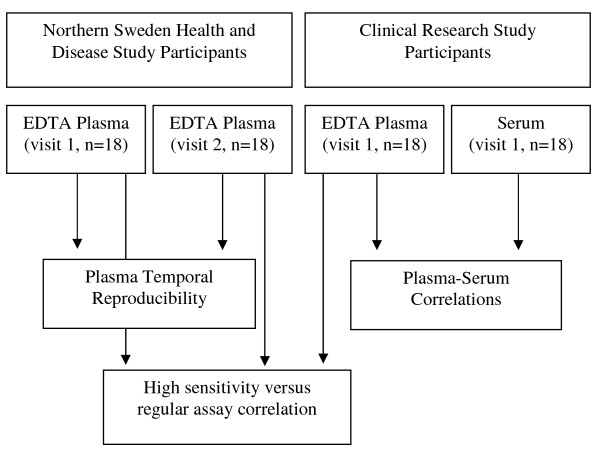
**Study Design**.

Eighteen female NSHDS participants between the ages of 42 and 62 who provided two blood samples at least 1-3 years apart (n = 36 samples (18 pairs)) are included in the present study to assess temporal reproducibility of cytokine measurements in EDTA plasma samples (Figure 1). Subjects were free of invasive cancer or other chronic diseases. All samples were run on the same well-plate to minimize laboratory batch effects. To estimate intra-batch coefficients of variation (CVs), duplicate EDTA plasma samples from the first blood donation for 8 subjects were also included on the same well-plate.

To assess the influence of sample type on the cytokine measurements, paired EDTA plasma and serum samples were collected from 18 postmenopausal women (age range: 75 to 86) who were participating in a clinical research study at the University of Umeå in Sweden (Figure 1). All subjects were free of cancer and cardiovascular disease. At enrollment, 20 mL non-fasting blood samples were collected into tubes containing no anticoagulant (serum) and tubes containing EDTA as an anti-coagulant (plasma). Sample processing was completed immediately after blood collection, under the same standardized procedure as the NSHDS samples, and serum and EDTA plasma fractions have since been stored at -80°C.

### Cytokine Measurements

We measured CRP, 22 cytokines and their soluble receptors (IL-1α, IL-1β, IL-1RA, IL-2, sIL-2R, IL-4, IL-5, IL-6, sIL-6R, IL-7, IL-8, IL-10, IL-12p40, IL-12p70, IL-13, IL-15, IL-17, TNFα, sTNF-R1, sTNF-R2, IFN-α, IFN-γ), and 8 growth factors (granulocyte macrophage colony stimulating factor (GM-CSF), epidermal growth factor (EGF), basic fibroblast growth factor (bFGF), G-CSF, HGF, vascular endothelial growth factor (VEGF), EGFR, and human epidermal growth factor receptor 2 (ErbB2)) using Luminex multiplex bead-based technology. For 11 biomarkers (IL-1β, IL-2, IL-4, IL-5, IL-6, IL-7, IL-8, IL-10, IL-13, TNFα, IFNγ), both regular [8] and high-sensitivity (hs) assays [13] were performed, resulting in a total of 42 measurements per sample. Thirteen biomarkers (hsIL-1β, hsIL-2, hsIL-4, hsIL-5, hsIL-6, hsIL-7, hsIL-8, hsIL-10, hsIL-12p70, hsIL-13, hsTNFα, hsIFNγ, and hsGM-CSF) were measured using a high-sensitivity (hs) kit from Linco/Millipore, CRP was measured using the CVD-2 kit from Linco/Millipore, EGFR and ErbB2 were measured with a kit developed in-house, and the other 27 markers were measured using a regular-sensitivity kit from Biosource International (Camarillo, CA). The assays were run in accordance with manufacturers' protocols and methods have been reported previously [9]. The lower limit of detection (LLD) and the coefficient of variation (CV) for each marker are presented in Table 1.

**Table 1:** Temporal reproducibility of EDTA plasma biomarkers measured by Luminex xMap™, NSHDS subjects ^a ^(n = 18 pairs)

Biomarker	LLD	First Sample			Second Sample			Intra-batch CV	ICC	(95% CI)
					
		% Above LLD	Median	(25%, 75%)	% Above LLD	Median	(25%, 75%)			
hsIL-1β (pg/mL)^b^	0.06	100	8.1	(5.5, 11.3)	100	8.0	(4.6, 10.1)	2.0%	0.73	(0.43-0.89)
hsIL-2 (pg/mL)^b^	0.16	100	28.8	(18.9, 39.1)	100	31.3	(20.8, 39.9)	2.7%	0.80	(0.56-0.92)
sIL-2R (pg/ml)^c^	30	100	652	(624, 789)	100	691	(624, 762)	1.0%	0.86	(0.68-0.95)
IL-4 (pg/mL)^c^	5	100	140	(114, 163)	100	120	(109, 143)	1.6%	0.00	(-0.45-0.45)
hsIL-4 (pg/mL)^b^	0.13	100	50.5	(31.4, 80.7)	100	42.4	(16.2, 83.0)	2.7%	0.70	(0.36-0.87)
hsIL-5 (pg/mL)^b^	0.01	100	3.4	(1.8, 4.6)	100	3.2	(1.8, 4.2)	2.5%	0.73	(0.41-0.89)
hsIL-6 (pg/mL)^b^	0.10	100	20.4	(15.9, 28.7)	100	18.4	(13.8, 24.3)	2.0%	0.81	(0.56-0.92)
sIL-6R (ng/mL)^c^	0.024	100	29.0	(26.5, 42.6)	100	29.8	(25.2, 36.4)	0.3%	0.69	(0.36-0.87)
hsIL-7 (pg/mL)^b^	0.12	100	10.1	(6.5, 15.7)	94.4	10.7	(6.1, 13.7)	3.3%	0.55	(0.14-0.80)
hsIL-8 (pg/mL)^b^	0.11	100	9.3	(6.4, 11.8)	100	8.3	(5.3, 10.5)	1.6%	0.86	(0.68-0.95)
hsIL-10 (pg/mL)^b^	0.15	100	40.7	(26.7, 57.2)	100	33.2	(15.8, 56.3)	2.1%	0.75	(0.46-0.90)
IL-12p40 (pg/mL)^c^	15	100	410	(379, 471)	100	413	(402, 447)	0.7%	0.89	(0.73-0.96)
hsIL-12p70 (pg/mL)^b^	0.11	100	32.3	(21.5, 46.5)	100	26.0	(11.8, 40.7)	2.1%	0.77	(0.50-0.91)
hsIL-13 (pg/mL)^b^	0.48	100	45.7	(30.5, 51.9)	100	39.6	(15.4, 50.2)	3.0%	0.81	(0.56-0.92)
CRP (μg/mL)^b^	0.000002	100	10.6	(3.1, 17.1)	100	8.5	(4.0, 16.9)	1.9%	0.76	(0.48-0.90)
IFNα (pg/mL)^c^	15	100	45.8	(41.1, 70.7)	100	50.2	(37.8, 80.7)	5.1%	0.53	(0.11-0.79)
IFNγ (pg/mL)^c^	5	100	175	(108, 199)	100	118	(95.3, 162)	1.8%	0.00	(-0.45-0.45)
hsIFNγ (pg/mL)^b^	0.29	100	78.3	(42.6, 99.4)	100	65.0	(43.4, 103)	2.2%	0.72	(0.41-0.89)
hsTNFα (pg/mL)^b^	0.05	100	8.0	(6.2, 11.2)	100	8.4	(5.8, 10.9)	1.9%	0.69	(0.36-0.87)
sTNF-R1 (ng/mL)^c^	0.015	100	1.3	(1.2, 1.8)	100	1.2	(0.9, 1.6)	1.7%	0.31	(-0.16-0.67)
sTNF-R2 (ng/mL)^c^	0.015	100	1.1	(0.72, 1.2)	100	0.9	(0.7, 1.1)	0.5%	0.68	(0.33-0.86)

**Growth Factors**										
EGFR (ng/mL)^d^	0.020	100	15.4	(14.1, 15.9)	100	15.3	(13.9, 16.6)	0.5%	0.93	(0.83-0.97)
ErbB2 (ng/mL)^d^	0.017	100	3.4	(2.9, 3.6)	100	3.2	(3.0, 3.8)	0.5%	0.63	(0.26-0.84)
hsGM-CSF (pg/mL)^b^	0.46	100	38.2	(29.6, 52.4)	100	33.5	(21.9, 39.9)	2.9%	0.47	(0.03-0.76)
G-CSF (pg/mL)^c^	15	100	129	(108, 167)	100	129	(108, 160)	6.4%	0.75	(0.46-0.90)
HGF (pg/mL)^c^	10	100	151	(101, 186)	100	196	(101, 278)	1.1%	0.74	(0.43-0.89)

*Note*: LLD = lower limit of detection as reported by the manufacturer, CVs are based on 8 blinded duplicates

^a ^Limited to the biomarkers for which more than 70% of all the samples were detectable. There were no extrapolated values for any of the markers included in the table except for 1 sample for IFNα. Sixteen markers are not included because more than 30% of samples were undetectable (IL-1β, IL-1RA, IL-5, IL-6, IL-17, VEGF), and/or a high percentage of samples were extrapolated below the lowest point on the standard curve (IL-1α, IL-2, IL-7, IL-8, IL-10, IL-13, IL-15, TNFα, EGF, bFGF).

^b ^Kits from Linco/Millipore^c ^Kit from Biosource

^d ^Kit developed in-house

### Statistical analyses

Cytokine fluorescence intensity (raw data) values were set to missing if they were below background. Values were log-transformed to reduce departures from the normal distribution. The intraclass correlation coefficient (ICC) was used to assess temporal reliability. The ICC estimates the fraction of the total variation (within- plus between-subject variation) due to between-subject variation [14]. The ICC can take on any value between zero and one; values close to zero are indicative of no correlation between the repeated measurements, while values close to one indicate high replicability of a given subject's measurements over time. A random effects one-way analysis of variance model was used to estimate the within- and between-subject variance components. ICCs were only calculated for markers that were above the lower limit of detection (LLD) in at least 70% of the samples. Furthermore, we did not estimate ICCs for markers which required extrapolation below the standard curve for a large percentage (> 40%) of the samples. While these markers could potentially be used in future epidemiological studies, the high percentage of missing or extrapolated values in the present study would have limited the interpretation of the ICCs. Our *a priori *criteria for inflammation markers to be considered for use in our epidemiological study of ovarian cancer risk was an ICC ≥ 0.55. A number of biomarkers with ICCs in this range have been shown to be consistent predictors of disease in epidemiological studies, such as postmenopausal endogenous estrogens (ICCs ranging from 0.5-0.7 over a 2-3 year period) [15-17], blood pressure (0.6 for systolic and diastolic over a 2-4 year period) [17,18], and serum cholesterol (0.6-0.7 over a 1-2 year period) [17,19].

To compare plasma and serum values, we computed the relative difference between each plasma/serum pair (plasma value minus serum value divided by plasma value) and report the median relative difference as a percentage. The Wilcoxon signed-rank test was used to test whether marker values were systematically higher in one of the sample types. Spearman correlation coefficients (r_s_) were calculated for EDTA plasma vs. serum samples for the 18 participants from the clinical research study.

For the 11 cytokines that were measured in EDTA plasma using both high-sensitivity and regular assays, Spearman correlation coefficients were calculated to assess the correlation between the assays. To increase the sample size for this analysis, we used all 54 plasma samples which had been measured by both assays. We were concerned that the high correlation between plasma samples collected annually from the NSHDS participants might bias the correlation coefficients. Thus, we also used a bootstrap method to randomly select one of the two plasma samples for each subject from the NSHDS study to create a group of 36 mutually independent samples (n = 18 of the 36 samples from NSHDS plus the 18 plasma samples from the clinical research study). We repeated this step 100 times and calculated the average Spearman correlation coefficient.

All study subjects provided written informed consent to participate in the study. The Regional Ethical Committee of the University of Umeå, Sweden, and the Swedish Data Inspection Board reviewed and approved this study.

## Results

### Temporal Reliability of EDTA Plasma Cytokines and Growth Factors

The mean age of the study subjects from the NSHDS study at their initial blood donation was 55.6 years and all subjects were of European descent. EDTA plasma samples from the first and second blood donations were stored for an average of 17.8 years and 15.6 years, respectively. The average time between blood donations was 2.1 years (range: 1.7-3.7 years). Four participants (22%) were current smokers at the time of first blood donation.

The lower limits of detection, percentage of samples above the lower limit of detection, and median cytokine values are shown in Table 1 for the 26 marker assays which yielded detectable values for more than 70% of the samples. Detection proportions and median values were similar for both visits (Table 1). Not included in the table are results for 16 marker assays which yielded undetectable values for more than 30% of the samples (IL-1β, IL-1RA, IL-5, IL-6, IL-17, VEGF) and/or required extrapolation below the lowest point on the standard curve for a high percentage of the samples (about 40% for IL-8 and bFGF and 60-95% for IL-1α, IL-2, IL-7, IL-10, IL-13, IL-15, TNFα, and EGF). Markers included in Table 1 had no extrapolated values except for one sample for IFNα.

CVs, ICCs, and 95% CIs for the ICCs are also shown in Table 1. These marker assays had satisfactorily low CVs (≤ 6.4%), indicating they can be reproducibly measured in EDTA plasma samples. Twenty-two of the 26 biomarker assays (hsIL-1β, hsIL-2, sIL-2R, hsIL-4, hsIL-5, hsIL-6, sIL-6R, hsIL-7, hsIL-8, hsIL-10, IL-12p40, hsIL-12p70, hsIL-13, CRP, IFNα, hsIFNγ, hsTNFα, sTNF-R2, EGFR, ErbB2, G-CSF, and HGF) had moderate to high ICCs, ranging from 0.53-0.89, which suggests that these markers are temporally reproducible in women over a 1-3 year period. Of the four marker assays with low ICCs (0.00 - 0.47), two markers (IL-4 and IFNγ) were also measured by alternative high-sensitivity assays which showed adequate ICCs in this study (hsIL-4 and hsIFNγ, Table 1).

### Comparison of EDTA Plasma versus Serum

Clinical research study subjects included in this report provided paired EDTA plasma and serum samples. The average age of the subjects at blood donation was 78.9 years and all subjects were of European descent. Samples were stored for an average of 10.9 years.

The percentage of samples above the LLD for EDTA plasma and serum are shown for the clinical research study participants in Table 2. The percentage of EDTA plasma samples that could be detected was the same for the clinical research subjects as for the NSHDS subjects. Values were only extrapolated below the standard curve for a small percentage (less than 14%) of samples for hsIL-5, IFNα, and IL-6R. Marker measurements were significantly higher in serum than EDTA plasma for sIL-6R, hsIL-7, hsIL-8, hsIL-12p70, sTNF-R2, EGFR, and HGF. Values were significantly lower in serum than EDTA plasma for IL-4 and IFNγ.

**Table 2:** Correlation between EDTA plasma versus serum biomarkers measured by Luminex xMap™, clinical research study subjects ^a ^(n = 18 pairs)

Biomarker	Plasma			Serum			Plasma vs. Serum Paired Relative Difference (%)		p-value (Wilcoxon Signed- Rank Test)	Spearman Correlation Coefficient (r_s_)	r_s _ p-value
				
	% Above LLD	Median	(25%, 75%)	% Above LLD	Median	(25%, 75%)	Median	(25%, 75%)			
**Cytokines**											
hsIL-1β (pg/mL)^b^	100	5.1	(3.4, 7.5)	94.4	3.5	(2.5, 8.9)	14	(-14, 42)	0.548	0.83	0.000
hsIL-2 (pg/mL)^b^	100	18.8	(9.8, 25.7)	100	17.4	(7.4, 35.6)	-2.3	(-39, 38)	0.799	0.69	0.002
sIL-2R (pg/ml)^c^	100	658	(602, 746)	100	660	(527, 831)	-4.7	(-17, 11)	0.459	0.72	0.001
IL-4 (pg/mL)^c^	100	141	(108, 181)	100	113	(96.4, 123)	19	(-3.3, 46)	0.009	-0.02	0.922
hsIL-4 (pg/mL)^b^	100	56.3	(27.9, 91.4)	100	63.0	(34.4, 108)	-15	(-41, 11)	0.159	0.28	0.265
hsIL-5 (pg/mL)^b^	100	2.3	(1.4, 3.1)	94.4	2.6	(0.8, 3.9)	11	(-16, 44)	0.459	0.77	0.000
hsIL-6 (pg/mL)^b^	100	20.3	(12.6, 35.7)	100	28.9	(13.3, 43.6)	-2.2	(-41, 7.6)	0.369	0.77	0.000
sIL-6R (ng/mL)^c^	100	27.5	(26.0, 34.6)	100	59.1	(47.9, 91.9)	-102	(-189, -68)	0.000	0.39	0.113
hsIL-7 (pg/mL)^b^	100	14	(9, 16.9)	94.4	14.8	(13.3, 18.6)	-32	(-80, 5.3)	0.008	0.64	0.006
hsIL-8 (pg/mL)^b^	100	10.3	(6, 21.5)	100	13.3	(7.9, 26.2)	-20	(-59, -1.8)	0.002	0.90	0.000
hsIL-10 (pg/mL)^b^	100	31.8	(19.9, 40.2)	100	25.5	(11.2, 48.8)	20	(-23, 30)	0.442	0.73	0.001
IL-12p40 (pg/mL)^c^	100	417	(361, 602)	100	417	(358, 537)	-0.4	(-6.9, 3.0)	0.393	0.96	0.000
hsIL-12p70 (pg/mL)^b^	100	20.8	(14.8, 32.6)	100	29.1	(16.5, 59.9)	-14	(-112, 1.3)	0.030	0.77	0.000
hsIL-13 (pg/mL)^b^	100	27.2	(19.6, 33.8)	100	32.6	(22.3, 53)	-20	(-56, 14)	0.057	0.66	0.003
CRP (μg/mL)^b^	100	20.5	(7.4, 35.7)	100	22.2	(8.6, 40.2)	-11	(-20, 4.1)	0.081	0.95	0.000
IFNα (pg/mL)^c^	100	42.7	(34.2, 53)	100	37.8	(34.2, 47.3)	4.1	(-20, 31)	0.318	-0.05	0.840
IFNγ (pg/mL)^c^	100	166	(125, 262)	100	83.0	(73.9, 91.1)	56	(20, 70)	0.000	-0.17	0.500
hsIFNγ (pg/mL)^b^	100	45.4	(25.4, 61)	94.4	38.4	(18.2, 78.8)	4.1	(-42, 40)	0.927	0.68	0.003
hsTNFα (pg/mL)^b^	100	10.7	(9.6, 13)	100	10.4	(8.6, 15)	-6.9	(-26, 8.8)	0.196	0.71	0.001
sTNF-R1 (ng/mL)^c^	100	1.5	(1.4, 1.8)	100	1.6	(1.1, 2.0)	-6.6	(-21, 14)	0.442	0.75	0.000
sTNF-R2 (ng/mL)^c^	100	1.4	(1.1, 1.8)	100	1.7	(1.2, 1.9)	-5.0	(-12, 0.1)	0.009	0.94	0.000

**Growth Factors**											
EGFR (ng/mL)^d^	100	13.7	(12.6, 14.8)	100	18.1	(17.1, 20.2)	-28	(-44, -18)	0.000	0.48	0.045
ErbB2 (ng/mL)^d^	100	3.6	(3.2, 4.1)	100	3.7	(3.1, 4.0)	-6.8	(-9.4, -1.6)	0.142	0.72	0.001
hsGM-CSF (pg/mL)^b^	100	21.3	(17.4, 31.3)	94.4	28.7	(19, 53.4)	-9.9	(-47, 8.1)	0.174	0.79	0.000
G-CSF (pg/mL)^c^	100	125	(99.6, 149)	100	91.4	(83, 123)	9.0	(-13, 34)	0.156	0.45	0.059
HGF (pg/mL)^c^	100	230	(205, 307)	100	380	(253, 687)	-50	(-165, -20)	0.000	0.72	0.001

*Note*: LLD = lower limit of detection

^a ^Limited to the biomarkers for which more than 70% of all the samples were detectable. Values were extrapolated below the standard curve for less than 14% of samples for hsIL-5, IFNα, and IL-6R, but were not extrapolated for any other markers included in the table. Sixteen markers are not included because more than 30% of samples were undetectable (IL-1β, IL-1RA, IL-5, IL-6, IL-17, VEGF), and/or a high percentage of the samples were extrapolated below the lowest point on the standard curve (IL-1α, IL-2, IL-7, IL-8, IL-10, IL-13, IL-15, TNFα, EGF, bFGF).^b ^Kits from Linco/Millipore^c ^Kit from Biosource

^d ^Kit developed in-house

Spearman correlation coefficients for EDTA plasma vs. serum measurements are also shown in Table 2. High correlations (r_s _ranging from 0.80 to 0.98) were observed for hsIL-1b, hsIL-8, IL-12p40, CRP, and sTNF-R2. Most other correlations were above 60%, although seven marker assays demonstrated low correlations (r_s _< 0.5) between paired plasma and serum samples (IL-4, hsIL-4, sIL-6R, IFNα, IFNγ, EGFR and G-CSF).

### Comparison of Regular and High-Sensitivity Assays

A subset of 11 cytokines (IL-1β, IL-2, IL-4, IL-5, IL-6, IL-7, IL-8, IL-10, IL-13, TNFα and IFNγ) were measured in EDTA plasma samples using both high-sensitivity and regular assays. Regular assays were limited in their ability to detect some of the markers as compared to high sensitivity assays and were more likely to require extrapolation below the standard curve (Table 3). Spearman correlation coefficients for the two assay types were low (r_s _< 0.5) for all markers, and all were non-significant except for IL-8. The average Spearman correlation coefficients estimated using the bootstrap method (data not shown) were not appreciably different from the coefficients estimated using all available plasma samples.

**Table 3:** Spearman correlations between the regular and high-sensitivity assays ^a^, NSHDS and clinical research study subjects combined (n = 54 samples)

Biomarker	LLD (pg/mL)		% of samples above LLD		**% of samples above LLD that required extrapolation **^b^		n samples used to compute r_s_	Spearman correlation coefficient (r_s_)	r_s _p-value
				
	Regular assay	High-sensitivity assay	Regular assay (%)	High-sensitivity assay (%)	Regular assay (%)	High-sensitivity assay (%)			
IL-1β	15	0.06	41	100	41	0	22	0.17	0.45
IL-2	6	0.16	52	100	64	0	28	0.18	0.36
IL-4	5	0.13	100	100	0	0	54	0.06	0.65
IL-5	3	0.01	0	100	N/A	2	N/A	N/A	N/A
IL-6	3	0.10	35	100	68	0	19	0.35	0.13
IL-7	10	0.12	50	98	93	0	27	0.11	0.59
IL-8	3	0.11	100	100	26	0	54	0.45	0.0007
IL-10	5	0.15	83	100	91	0	45	0.23	0.13
IL-13	10	0.48	98	100	81	0	53	-0.13	0.37
TNFα	10	0.05	100	100	66	0	54	0.15	0.28
IFNγ	5	0.29	100	100	0	0	54	0.08	0.56

*Note*: All available plasma samples were used for these analyses, i.e., a maximum of 36 samples for the NSHDS and 18 samples for the clinical research subjects.

^a ^Regular assay kit from Biosource and high-sensitivity (hs) assay kit from Linco/Millipore

^b ^Values were extrapolated beyond the lowest point on the standard curve if their florescence intensity reading was above background florescence intensity. If florescence intensity was less than background florescence intensity, values were considered to be below the lower limit of detection.

## Discussion

We found that 22 cytokines and growth factors were detectable in over 70% of EDTA plasma samples and had ICCs of at least 0.53, indicating that for these markers, a single measurement is representative of an individual's average level (at least over a 2-year period), relative to other individuals. Of the 21 marker assays that had insufficient ICCs or which yielded undetectable values for more than 30% of the samples, 11 markers (IL-1β, IL-2, IL-4, IL-5, IL-6, IL-7, IL-8, IL-10, IL-13, TNFα, and IFNγ) could be measured reliably using an alternative high-sensitivity assay.

Our ICC estimates apply to a nested case-control study in which samples from cases and their matched controls are measured in the same laboratory batch. Including samples of case-control matched sets in the same batch has the advantage of controlling for between-batch variability, and is the usual approach for most biomarker studies within cohorts. A limitation of this study is that our sample size was small, which resulted in wide confidence intervals for the ICC estimates.

Several studies have reported that cytokines may be sensitive to sample type, though the majority of these studies compared serum vs. citrate or heparin plasma [20-24]. In the present study, we observed that EDTA plasma vs. serum measurements were moderately to highly correlated (r > 0.60) for 21 of the 26 biomarker measurements. Median biomarker values were generally similar for serum and EDTA plasma samples, except for sIL-6R, hsIL-7, hsIL-8, hsIL-12p70, sTNF-R2, EGFR, and HGF, which were higher in serum, and IL-4 and IFNγ, which were higher in EDTA plasma.

For markers for which both regular and high-sensitivity assay kits were available, the high-sensitivity assays were markedly superior to the regular-sensitivity assays, both in the percentage of samples that were detectable and in the reproducibility of the measurements over time. The correlations between the regular and high-sensitivity assay measurements were very low. Variation between assay kits is commonly reported [25], and is likely to result from technical differences in the design of the assay [26-28]. For example, the use of different antibodies between kits could result in lower detection of a cytokine if one antibody recognizes an epitope that is commonly bound to a soluble receptor or a serum protein (e.g. albumin), or is present in a dimeric or trimeric form [29]. For the purposes of this study, kits were selected because they had sufficient assay sensitivity (minimum detectable concentrations), precision (intra- and inter-batch variation), and accuracy (% recovery of spiked serum samples) according to validation data provided by the manufacturers or through laboratory validation of the in-house assays. Based on the sensitivity and recovery-rate data provided by the manufacturers, we expected that the Millipore high-sensitivity assay would substantially improve detection rates over the regular sensitivity assay. Other reports on the validity of various multiplex assays in relation to other platforms (e.g. ELISAs or RIAs) are available for consideration when selecting a kit [20,27,30-32]. Studies that compared Luminex to ELISA in healthy subjects have reported low (for IL-6 and TNFα in two out of three studies) to high correlations depending on the cytokine of interest [8,20,33].

For most cytokines, median values were similar or slightly lower (e.g., hsIL-1β, hsIL-2, hsIL-10, hsIL-13, hsIFNγ, hsGM-CSF) in samples from the clinical research study subjects (Table 2) versus the NSHDS subjects (Table 1). On the other hand, median CRP and HGF values were almost 50% higher in the clinical research subjects than the NSHDS subjects. It is unlikely that this difference between study groups is due to differences in storage time (on average 5-10 years shorter for clinical research subjects) or sample processing, since both of these markers are known to be stable during long term storage, freeze thaw cycles, and under different sample processing conditions [34-36]; rather it is likely reflective of differences in participant characteristics, in particular, age (clinical research subjects were an average of 23 years older).

Samples with florescence intensity values below background may actually have low cytokine values and could potentially be imputed for epidemiological studies. CVs, ICCs, and Spearman correlation coefficients did not differ when we set the florescence intensity values that were below background to zero rather than missing. Investigators should examine the effect of classifying subjects with values below background as having low cytokine values vs. missing values on measures of association and report any observed differences.

We previously evaluated the temporal reliability of these marker assays in *serum *samples from women in the prospective New York University Women's Health Study cohort. Six markers met our a priori criteria, ie detectable in over 40% of samples and ICC threshold of 0.55, in the present study of EDTA plasma that did not make this cutoff in the serum reliability study: sIL-6R (ICC = 0.52 in the serum study), and sIL-2R, IL-15, hsIFNγ, G-CSF, and bFGF (not detectable in over 40% of the serum samples) [9]. Two markers that met the ICC cutoff value of 0.55 in the previous serum study (IL-1RA ICC: 0.57 and sTNF-R1 ICC: 0.68) did not meet this criterion in the present EDTA plasma study. This suggests that the ICCs for these assays may be different for serum and plasma, though the sample size of the current plasma study was small, and thus the confidence intervals were fairly wide. Although we did not have information on a number of potential covariates of interest for the present study (nor the power to evaluate the influence of these covariates on cytokines given our small sample size), the report on *serum *cytokines found that adjustment for covariates (age at blood donation, order of blood donation, blood storage time, menopausal status, phase of menstrual cycle (for premenopausal women), BMI, ethnicity, medication use, alcohol consumption and smoking status) did not change the ICC estimates appreciably [9].

We found that 22 out of the 31 biomarkers evaluated in the current report were detectable in a majority of samples, temporally reliable over an average of 2 years (ICC ≥ 0.53), and measured reproducibly (CV <10%). These results suggest that a single measurement of these biomarkers may be used in epidemiologic studies using banked EDTA plasma samples collected before disease diagnosis to evaluate risk.

## Competing interests

The authors declare that they have no competing interests.

## Authors' contributions

TVC participated in the design of the study, performed the statistical analysis, and drafted the manuscript. AZJ, KLK, AAA, and EL participated in the design of the study and the analysis and interpretation of the data, and made substantial revisions to the manuscript for important intellectual content. EL also provided the plasma and serum samples along with GH, who participated in the study design, interpretation of the data and revision of the manuscript. AEL supervised the multiplex inflammation marker assays, participated in the interpretation of the data, and contributed revisions to the manuscript. BN and AM carried out the multiplex inflammation marker assays and were involved in revision of the manuscript. AI and NO were involved in the interpretation of the data and revision of the manuscript. All authors approved the manuscript.
